# Chimeric antigen receptor-engineered natural killer cells for cancer immunotherapy

**DOI:** 10.1186/s13045-020-00998-9

**Published:** 2020-12-07

**Authors:** Ahmet Yilmaz, Hanwei Cui, Michael A. Caligiuri, Jianhua Yu

**Affiliations:** 1 The Ohio State University Comprehensive Cancer Center, Columbus, OH 43210 USA; 2 Department of Hematology and Hematopoietic Cell Transplantation, City of Hope National Medical Center, 1500 E. Duarte Road, KCRB, Bldg. 158, 3rd Floor, Room 3017, Los Angeles, CA 91010 USA; 3 Hematologic Malignancies and Stem Cell Transplantation Institute, City of Hope National Medical Center, Los Angeles, CA 91010 USA; 4 Department of Immuno-Oncology, City of Hope Beckman Research Institute, Los Angeles, CA 91010 USA; 5 City of Hope Comprehensive Cancer Center and Beckman Research Institute, Los Angeles, CA 91010 USA

**Keywords:** Chimeric antigen receptor, Natural killer cells, T cells, Cancer immunotherapy

## Abstract

Natural killer (NK) cells are a critical component of the innate immune system. Chimeric antigen receptors (CARs) re-direct NK cells toward tumor cells carrying corresponding antigens, creating major opportunities in the fight against cancer. CAR NK cells have the potential for use as universal CAR cells without the need for human leukocyte antigen matching or prior exposure to tumor-associated antigens. Exciting data from recent clinical trials have renewed interest in the field of cancer immunotherapy due to the potential of CAR NK cells in the production of “off-the-shelf” anti-cancer immunotherapeutic products. Here, we provide an up-to-date comprehensive overview of the recent advancements in key areas of CAR NK cell research and identify under-investigated research areas. We summarize improvements in CAR design and structure, advantages and disadvantages of using CAR NK cells as an alternative to CAR T cell therapy, and list sources to obtain NK cells. In addition, we provide a list of tumor-associated antigens targeted by CAR NK cells and detail challenges in expanding and transducing NK cells for CAR production. We additionally discuss barriers to effective treatment and suggest solutions to improve CAR NK cell function, proliferation, persistence, therapeutic effectiveness, and safety in solid and liquid tumors.

## Background

Natural killer (NK) cells are large granular lymphocytes with surface markers CD3^−^CD56^+^NKp46^+^ in humans. They have been recognized as a subset of innate lymphoid cells (ILCs) that lack antigen receptors with recombination activating gene (RAG)-dependent rearrangement [1, 2]. There are three groups of ILCs (Groups 1–3), each with distinct functions and expression profiles of key transcription factors. NK cells are included in Group 1 ILCs, which also include innate lymphoid cell 1 (ILC1). Both NK cells and ILC1 depend on the transcription factor T-bet for their proper function and development, and secrete interferon-γ (IFN-γ) rather than type 2 cytokines (IL-5 and IL-13), IL-17, or IL-22 [3]. The development of NK cells involves bone marrow and extra-medullary sites such as tonsils, uterus, and liver [4–6]. The NK cell developmental process is initiated in the bone marrow microenvironment where hematopoietic stem cells differentiate into common lymphoid progenitors (CLPs) which can give rise to all subtypes of lymphoid cells. CLPs first develop into NK progenitor cells, and then differentiate into immature and finally mature or resident NK cells. These developmental processes may occur in different organs or tissues [7–9].

NK cells are able to recognize and kill “stressed cells” including cells infected with viruses, allogeneic cells, and tumor cells without prior sensitization in a non-HLA (human leukocyte antigen)-restricted manner. The effector functions in NK cells are regulated through a balance of signals delivered by activating and inhibitory receptors as well as cytokines such as IL-2, IL-12, IL-15, or IL-18 [10]. The inhibitory receptors, including inhibitory killer immunoglobulin receptors (KIRs) and NKG2A/CD94 in humans, keep NK cells in a quiescent state through the recognition of constitutively expressed self-MHC class I molecules on healthy cells. A variety of activating receptors are expressed on NK cells, exemplified by NKG2D, CD16, natural cytotoxicity receptors (NCRs), and activating KIRs. The activating receptors contribute to NK cell activation via recognition of host-derived or pathogen-encoded ligands or antibodies [11, 12].

NK cells play critical roles against tumor initiation and metastasis [13, 14]. Their activation by tumor cells, which depends on the type of ligands on the surface of the tumor cells, can be accomplished via several mechanisms. The MHC-I molecules on target cells, which inhibit NK cell function through binding to inhibitory receptors on NK cells, are often reduced or lost in tumors (missing-self recognition). Activation of NK cells can also be triggered by upregulation of ligands on tumor cells by certain therapeutic agents [15–17].

Activated NK cells are capable of eliminating tumor cells through direct cell cytotoxicity and/or production of pro-inflammatory cytokines. They can directly release perforin and granzymes to kill tumor cells. They can also mediate antibody-dependent cellular cytotoxicity (ADCC) via the membrane receptor CD16, or apoptotic pathways mediated by Fas ligand (FasL) or TNF-related apoptosis-inducing ligand (TRAIL) [18]. In addition, cytokines and chemokines such as IFN-γ and granulocyte macrophage colony stimulating factor (GM-CSF) produced by NK cells can modulate immune responses of other cells. For example, IFN-γ helps in activation of CD8 + T cells, monocytes, and macrophages [4, 19, 20].

Although NK cells are able to recognize and lyse tumor cells, the tumor cells develop their own mechanisms to evade recognition by NK cells or repress NK cell function. For example, tumor cells can produce immunosuppressive cytokines such as IL-10 or transforming growth factor-beta (TGF-β) to repress NK cell function [21, 22]. They may also reduce expression of tumor-associated antigens (TAAs) or elevate expression of MHC class I-related molecules to inactivate NK cells [23]. Engineering NK cells to express a chimeric antigen receptor (CAR) may overcome immune evasion. Although several approaches including CAR T cells, checkpoint inhibitors, antibodies, antibody–drug conjugates, and tumor vaccinations are being investigated [24, 25], development of “off-the-shelf” CAR-modified NK cells is an especially promising and unique approach in developing sustainable anti-cancer immunotherapeutic products.

### Chimeric antigen receptors (CARs): structure and function

A CAR is a functional hybrid antigen receptor usually combining variable regions of an antibody with the constant region of a T cell receptor (TCR), grafting an immune cell with the specificity for TAA expressed on tumor cells [26]. A typical CAR is composed of a signal peptide, single-chain antibody variable fragment (scFv), and transmembrane and intracellular signaling domains. A linker combines the variable regions of heavy and light chains of an antibody molecule. A hinge, also called spacer, connects the scFv to the transmembrane domain. scFv is derived from a tumor-specific antibody and can target any antigen expressed on tumor cell surface [27]. In some CARs, a ligand of a tumor antigen is used [28, 29]. An intracellular signaling domain is derived from immunoreceptor tyrosine-based activation motifs (ITAMs) of TCR or a cytoplasmic domain of other activating receptors. Phosphorylation of ITAMs recruits adapter molecules, stimulates downstream pathways, and activates immune cells [30, 73].

The first-generation CARs contained only the CD3ζ chain, the common signal transducing subunit of TCR, or FcRγ subunits as the intracellular signaling domain [31–33]. They were not effective in initiating T cell activation or tumor elimination as they lacked co-stimulatory signaling required for full T cell activation. To improve the immune cell response to tumor cells, second-generation CARs were created by adding one of the T cell co-stimulatory molecules such as CD28, 4-1BB (CD137), ICOS, or OX40 (CD134) in addition to CD3ζ. The third-generation CARs include three signaling domains (for example, CD3ζ and two co-stimulatory domains) [34, 35]. There is no consistent evidence suggesting increased performance in the third—compared to the second—generation CARs. The type of co-stimulatory domain used can dramatically influence effector functions mediated by the CAR. For example, 4-1BB co-activation results in development of T memory cell characteristics, whereas CD28 co-activation results in enhanced T cell activation and expansion [36].

### Generation of vectors for CARs

CARs can be generated against any of the surface antigens expressed on tumor cells. However, the target should have a specific antigen density to avoid "on-target, off-tumor" effects. Studies in T cells have shown that target density between 200 and 2000 copies per cell is optimal to avoid damage to the normal cells [37]. Generation of a CAR usually starts with an scFv derived from an antibody, composed of heavy and light chains [38]. Recently, nanobodies composed only of heavy chains are being utilized for CAR NK cell production [39]. These single-chain antibodies not only possess specificity comparable to scFvs but also are much smaller in size, facilitating transduction. However, their use in CAR NK cell production is currently at the experimental stage.

**Fig. 1:**
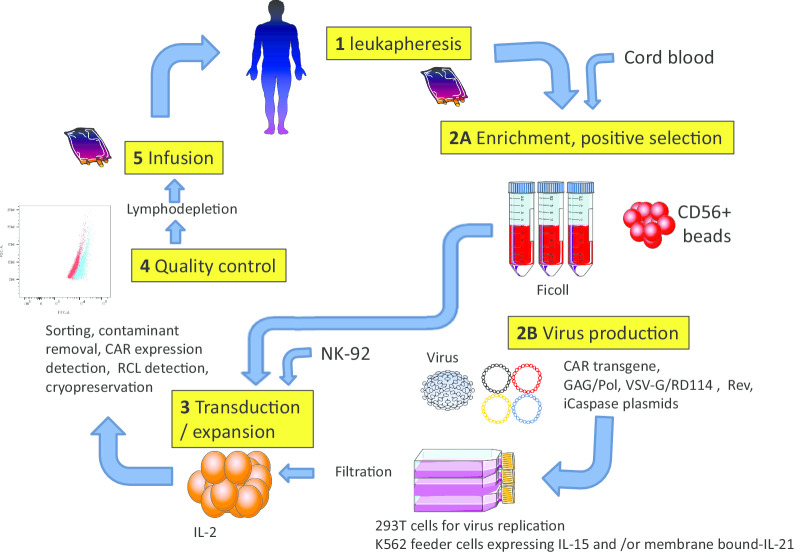
Representative steps in CAR NK cell manufacturing for immunotherapy, *RCL/RCR* replication competent lentivirus/retrovirus measured by qPCR for detection of DNA encoding a viral envelope

Usually, a signal peptide is introduced in the front of an scFv. A linker that connects heavy and light chains usually contains the (G_4_S)_3_ or (G_4_S)_5_ signature that provides flexibility for accurate folding of connected protein domains [40, 41]. A spacer, also referred to as a hinge, usually derived from IgG, CD8α, or CD28, plays a critical role in specificity of the CAR-bearing immune cells [41]. Adjusting the length of spacer may help optimize the distance between CAR-bearing cells and target cells for an optimal immunologic synapse (IS) formation and specificity. Use of CAR T cells created using spacers with suboptimal length results in off-target effects. The downstream CAR component(s) are co-stimulatory domain(s) that can be shared between CAR NK and CAR T cells [42, 43], followed by CD3ζ. With the rapid development of DNA synthesis technology, many CAR sequences are now synthesized by a vendor if the DNA sequences encoding the scFv are known. All CAR components or a cassette are commonly cloned into a retrovirus- or lentivirus-based expression vector, which is then used to transduce primary NK cells or NK cell lines [44] (Fig. 1). In some instances, an inducible iCaspase can be incorporated as a safety switch for use in case of adverse reactions [10]. Different CAR designs and detailed engineering approaches have been reviewed elsewhere [45].

### CAR T cells vs. CAR NK cells for cancer immunotherapy

CAR T cells targeting CD19 or other B cell markers such as CD20 have achieved remarkable responses in patients with B cell malignancies including chronic lymphocytic leukemia (CLL), acute lymphoblastic leukemia (ALL), and lymphoma [26, 46–48]. CTL019 (Kymriah, tisagenlecleucel) marketed by Novartis achieved 82.5% overall remission rate (ORR), which includes 40 complete remission (CR) and 12 CR with incomplete hematologic recovery (CRi) in 63 patients with ALL. A biologic license application for Kymriah was assessed based on B2202, a single-arm, open label, multi-center phase 2 study including 107 patients who signed consent. There were 19 patients who were not eligible due to death before the study began, unacceptable apheresis, incomplete screening before the cutoff date, or loss of eligibility for other reasons [49]. Additional twenty patients from the remaining 88 were further excluded due to adverse events, manufacturing failure, death, or pending infusion. The remaining 68 patients were included in the safety set, but five were further excluded from the efficacy set because the products were manufactured in Germany, and thus, 63 patients were included in the ORR calculation above. The ORR of 82.5% was an impressive outcome compared to less than 20% of the standard treatment, and thus, the product received an expedited approval by the US Food and Drug Administration (FDA) [36, 49, 50]. The intent-to-treatment (ITT) rate, as defined by the number of patients with ORR divided by the number of patients consented, was 48.5% (52/107). In the Yescarta study, 111 patients were enrolled, but 10 withdrew [51]. Objective response rate was 72% (51% for advanced cases). In the treatment of DLBCL, CR rates were higher in the Yescarta study compared to the Kymriah study (72% vs. 50%). It is unknown whether this difference was influenced by differences in the number of cells infused (60–600 million cells were infused if the body weight was greater than 50 kg in the Kymriah study and 2 million cells per kg were infused in the Yescarta study).

CD19-CAR T cells are the first CAR T cell-based drug approved by the FDA. Studies to develop CAR T cells for treating other tumor types including solid tumors are increasing [52–55]. However, there have been reports of serious side effects in the clinic associated with CAR T cell therapy including cytokine release syndrome (CRS), “on-target, off-tumor” toxicity, neurologic symptoms, graft vs. host disease (GvHD) if used in an allogeneic setting, and tumor lysis syndrome. For example, 94% of the patients in the Yescarta study and 74% in the Kymriah study had CRS and at least four two patients in each study died from CRS [51]. CRS is a rapid and massive release of inflammatory cytokines such as IFN-γ, TNF-α, and IL-6 into circulation [47]. It can present with symptoms such as high fever, malaise, fatigue, myalgia, nausea, anorexia, tachycardia/hypotension, capillary leak, or disseminated intravascular coagulation (reviewed by Bonifant et al. (2016) [56, 57]. Other studies have also shown that CRS by CAR T cells can be life-threatening [56–58]. In addition, CAR T cells may attack not only tumor but also normal cells, resulting in on-target, off-tumor toxicity. For example, HER2-specific CAR T treatment resulted in acute respiratory failure and death in a patient with metastatic colon cancer possibly due to low levels of HER2/neu expressed in the pulmonary parenchyma or vasculature [59]. Severe neurological toxicities such as acute cerebral edema resulting in brain death a few hours after initial appearance of neurological symptoms have also been reported in treatment with CD19-CAR T cells [60]. In the separate Kymriah and Yescarta studies, although, respectively, noted in 72% and 87% of the patients treated with CAR T cells, no deaths were attributed to neurotoxicity. CAR T cells obtained from allogeneic sources may also result in GvHD [61]. Disabling the endogenous TCR has the potential to avoid or lower GvHD risk. Endogenous TCR has been disabled using recent techniques such as CRISPR/Cas9, TALEN, zinc finger endonucleases, and receptor blockage using an array of proteins [61]. Some of these techniques are currently being evaluated for clinical use.

Compared to T cells, NK cells offer many advantages for cancer immunotherapy (Table 1). For example, it has been reported that allogeneic NK cells do not need complete HLA matching and cause little or no GvHD compared to allogeneic T cells [61–63]. In fact, there is experimental data in animals showing that NK cells not only do not cause GvHD but also prevent it in some cases by inhibiting activated alloreactive T cells while retaining graft vs. tumor effects [63]. On the contrary, CAR T cells can cause GvHD even when HLA-matched as minor mismatches may still be provocative [64]. Additionally, the cytokine levels produced by NK cell infusions are moderate [65] compared to the levels seen in life-threatening cases of CRS observed in CAR T cell trials [61].

**Table 1:** Comparison of clinical efficacy and future prospects in CAR T cell versus CAR NK cell cancer immunotherapy^a,b^

Parameter	CAR T	Primary CAR NK	CAR NK-92
Source	PB	PB or UCB	Cell line
Need for in vitro expansion before infusion	Yes	Yes	No
Pre-activation and differentiation	Needs CD3/CD28 stimulation	Requires feeder cells and/or IL-2 or other cytokines for optimal growth	Requires IL-2 for optimal growth
Viral transduction efficiency	Variable	Variable	High transduction efficiency
Side effects			
Neurotoxicity	Yes (reported in 58–87% of the patients in the Kymriah and Yescarta clinical trials)	No	No
CRS	Yes (reported in 74–94% of the patients in the Kymriah and Yescarta clinical trials)	No	No
Long-term side effects	Yes, CAR T cells can persist in circulation for years	Limited in vivo survival without IL-15 and persistent growth for over a year with incorporated IL-15	Short persistence, the cell line needs to be irradiated prior to infusion
Killing activity	Kills only tumor cells carrying TAA in an MHC-independent manner; produces slow response	Kills tumor cells regardless of their MHC status with some preference in killing tumor cells lacking MHC-I; quick response without prior activation	Similar to primary CAR NK
ADCC activity	No	Yes, due to CD16 expression	Similar to primary CAR NK when NK-92 CAR NK cells are transduced with CD16
Prospects for clinical use	Impressive clinical outcomes have already been reported; CAR T treatment has been approved by the FDA (Kymriah for ALL and Kymriah and Yescarta for DLBCL [51]	Being developed and promising	Being developed and combined with other agents
"Serial" killing ability	Yes	Yes, one NK cell can kill up to 5 tumor cells [220]	Unknown because cells are irradiated before infusion
Production efficiency	Low; needs to be prepared individually for each patient and requires coordination between production facility and clinic^a^	High due to donor availability and the off-the-shelf potential	High; unlimited expansion potential of the cell line
Cost of treatment	Expensive; approximately $373,000 USD [221]	Relatively less expensive due to off-the-shelf potential	Relatively less expensive than CAR T and primary CAR NK cells due to unlimited expansion potential
Potential for production of "off-the-shelf" anti-cancer products	Low to moderate; TCR knock-out studies are ongoing, but disabling TCR may lower T cell activity and/or survival and may be technically challenging	High; UCB NK cells may have better survival rates than PB NK cells after thawing or infusion	Very high; unlimited expansion potential

^a^10% of the patients died or were dropped and 80% needed bridge chemotherapy while waiting for the CAR T cell product during Kymriah and Yescarta clinical trials, complicating interpretation of the results [51]. This table has been partially adopted from Kurlander (2017), O’Leary (2017) [49, 51], and Klingemann (2014) [65]

^b^*DLBCL* diffuse large B-cell lymphoma, *PB* peripheral blood, *UCB* umbilical cord blood

NK cells have a limited life span in circulation and do not produce memory cells except for some viruses. Therefore, a primary-NK cell-based CAR approach may not need to be modified to include a suicide gene as a safety switch [66]. A further potential advantage of using CAR NK cells is that, unlike CAR T cells, they can be derived from umbilical cord blood (UCB) or cell lines such as NK-92, both of which have the potential for use as an “off-the-shelf” therapeutic product [61].

The safety of CAR NK-92 cells was tested in acute myeloid leukemia (AML) patients in a recent “first-in-man” clinical trial targeting CD33 [67]. Although the CAR NK-92 cells did not seem significantly beneficial in that particular study, injection of approximately five billion irradiated cells per patient did not produce any significant adverse side effects, suggesting that injection with a large number of NK-92 cells may be safe. A recent clinical trial (NCT03056339) produced exciting evidence supporting the potential for production of “off-the-shelf” NK cells with significant benefits in relapsed or refractory CD19-positive lymphoma and leukemia [68]. HLA-mismatched NK cells isolated from UCB and transfected with a retrovirus-based vector expressing anti-CD19 CAR, iCaspase-9, and IL-15 were administered in 11 patients with CLL or non-Hodgkin’s lymphoma. Nine patients received haplo-mismatched CAR NK cells and 2 patients received completely mismatched CAR NK cells.  Seven patients had a complete response and one had a partial response with remission of the Richter’s transformation component. These patients had relapsed/refractory tumors and were heavily pre-treated using conventional methods before starting CAR NK cell therapy. The patients had a median of four lines of therapies before beginning the CAR NK cell treatment. Five were CLL patients who failed to respond to ibrutinib and other therapies. Six were lymphoma patients, four of whom failed to respond to autologous hematopoietic stem cell transplantation (HSCT), and two had refractory disease. All but one patient had at least two prior relapses. An objective response of eight patients (73%) was observed during a follow-up period of 13.8 months. In all eight patients, a response was seen in the first month of the CAR NK treatment. An additional patient had a complete response, but his response was not attributed to CAR NK cell therapy. It was possible to bridge five of the eight patients who responded CAR NK therapy to HSCT, rituximab, venetoclax or lenalidomide treatment after CAR NK-cell-induced remission. None of the side effects seen with CAR T cells were noted. Excluding transient myelotoxicity, there was no neurotoxicity, elevation in IL-6 levels, CRS, or GvHD. The infused CAR NK cells expanded and persisted in vivo for at least 12 months [68].

### Sources to obtain NK cells for immunotherapy

NK cell lines as well as primary NK cells from peripheral blood (PB), UCB, or induced pluripotent stem cells (iPSCs) have been used for CAR NK cell production. Several NK cell lines such as NK-92, NKG, YT, NK-YS, HANK-1, YTS, and NKL are commercially available [69]. NK-92 is the most widely used cell line and has already entered into clinical trials [70]. In comparison to PB NK cells, NK-92 cells are easier to expand and transduce and do not carry any contaminating T cells that may cause GvHD [71]. In addition, their production is inexpensive and easy to standardize, and they do not express fewer inhibitory KIRs that restrict NK effector functions [72, 73]. However, there are also several disadvantages associated with the use of NK-92 cells. For example, the cells must be irradiated before infusion into patients to prevent unrestricted cell growth, thereby limiting their capacity for in vivo expansion. Additionally, the NK-92 cell line carries Epstein–Barr virus and has an abnormal genome.

NK cells constitute only about 10% of the PB lymphocytes and thus need expansion in vitro to obtain a sufficient quantity for a therapeutic effect. Various methods to enhance expansion of NK cells isolated from PB have been detailed below in the "Challenges in expanding CAR NK cells" section. NK cells from PB, and consequently CAR NK cells produced from PB, are potent killers of tumor cells as they are fully matured [74, 75]. Another well-studied source of primary NK cells is UCB, which may offer several advantages. Approximately 15 to 30% of UCB lymphocytes are NK cells, which can result in more than NK cells found in PB [76, 77]. There are less contaminating T cells in UCB compared to PB, which reduces the risk of GvHD [78–80]. In addition, NK cells in UCB may be less likely to undergo rejection by the patient compared to PB NK cells. However, NK cells isolated from UCB are immunologically immature and initiate only a weak immune response against target cells after being activated by stimuli such as cytokines or alloantigens [81]. Therefore, CAR NK cells derived from UCB may be less cytotoxic than those derived from PB [82]. UCB is easy to collect with no harm to the mother or child and almost never contaminated with Epstein–Barr virus or cytomegalovirus.

A recent study showed that CD19-CAR NK cells isolated from UCB transduced to express IL-15 and an iCaspase-9-based suicide gene had effective and persistent anti-tumor activity in NOD scid gamma (NSG) mice [83]. The incorporated IL-15 dramatically increased anti-tumor activity in the CD19-CAR NK cells. This study has been translated into a clinical trial with impressive outcomes [68]. Other less common sources for obtaining NK cells include pluripotent stem cells (human embryonic stem cells or iPSCs or adult stem cells (CD34+ hematopoietic stem cells) [84–88]. Recently, Li et al*.* (2018) utilized NK cells derived from human iPSC with a mesothelin-CAR modification as a potential source for "off-the-shelf" CAR NK cells for anti-cancer immunotherapy [89].

### Targets for CAR NK cells

The choice of targets for CARs depends on the type of tumor studied. Several proteins expressed by tumor cells are being targeted in preclinical or clinical studies. In lymphoid malignancies, the most widely targeted cell surface proteins include CD19 and CD20 [90–97]. CAR NK cells for multiple myeloma (MM) have targeted CD138 and CS1 [44, 98]. CS1 is a cell surface glycoprotein highly and ubiquitously expressed on the surface of myeloma cells [99]. In our previous studies, we found that CS1-CAR NK cells were able to target MM cells and eradicate MM both in vitro and in vivo [44]. MM cancer stem cell-like cells (CSCs) expressed significantly higher levels of CS1 than any other cell type, making them especially vulnerable to being eradicated by CS1-CAR NK cells [100]. Thus, CS1-CAR NK cells may prevent MM relapse via targeting and eliminating MM CSCs. CD22, important in B cell trafficking, can be targeted for treatment of B-cell lymphoma [101]. CD7, important in lymphoid development and B/T cell cross talk, can be targeted in ALL, whereas CD33, a lectin inhibiting multiple cellular functions, can be targeted in AML [102].

As for solid tumors, a set of targets have been evaluated. A well-known target is HER2 that is up-regulated in several cancers including breast, colon, and ovarian cancers [103]. HER2-CAR cells using PB NK cells or NK-92 cells have been used to treat breast and ovarian cancers and neuroblastoma [104–108]. GD2 has been targeted for treating neuroblastoma using CAR NK-92 cells [109, 110]. GD2-CAR NK-92 cells effectively lyse multi-drug resistant neuroblastoma cell lines and show significant anti-tumor activity in xenograft mouse models [110]. Additional antigens being explored as CAR NK cell targets in solid tumors include EGFR, GPA7, CD244, and EpCAM [44, 111–114].

EGFR is one of the most frequently amplified genes in glioblastoma, the most frequent and lethal type of brain tumor [115]. Approximately 20–40% of the EGFR-amplified tumors harbor the EGFR variant III (EGFRvIII). In our previous work, we found that EGFR-CAR NK cells targeted glioblastoma with both wild-type EGFR and EGFRvIII. EGFR-CAR NK cells were strongly cytotoxic, inhibited glioblastoma growth, and prolonged survival in tumor-bearing mice [112]. Use of CAR NK cells in combination with oncolytic viruses has been tested to eliminate solid tumors. Combination of EGFR-CAR NK-92 cells with herpes simplex virus 1-based oncolytic virus (oHSV-1) results in increased efficiency in killing a breast cancer cell line engrafted into the brain [116].

Additional targets being evaluated in recent clinical trials include a prostate-specific membrane antigen (PSMA) expressed over 100 times higher in prostate tumor tissues than normal prostate and the tumor differentiation antigen mesothelin in ovarian cancer [117, 118]. CAR NK cells and CAR NKT cells have been reported to target MUC1, a glycoprotein that creates a significant barrier preventing drugs from binding to their targets on cancer cells [119]. ROBO1, important in neuronal development, is being evaluated for treatment of pancreatic cancer [120].

### Challenges in CAR NK production

#### Challenges in transducing CAR NK cells

Several methods including electroporation and viral transduction methods have not achieved full transduction efficiency in CAR NK cell production (Table 2). Major groups of viruses tested for transduction include retro-, lenti-, vaccinia-, and adenoviruses. Vaccinia- and adenoviruses are not well suited for NK cell transduction as they may change the phenotype of the cells or may not bind receptors on NK cells [121]. In addition, transduction with adenoviruses is transient as they rarely integrate into the host genome [122]. Although transduction with retro- and lentiviral vectors has the advantage of achieving stable transgene expression, they may induce oncogenic transformation due to their potential for insertional mutagenesis [66]. For example, leukemia due to insertional mutagenesis has been reported in patients undergoing gene therapy with CD34+ cells transduced with retroviruses to restore IL-2 receptor expression [123]. Several studies have reported that insertion of retroviruses is more common in transcriptionally active than silent genes and transcription factor binding sites and thus may not be a completely random event. Likewise, lentiviruses also show strong preference for integrating into the transcriptionally active genes [124, 125]. Retroviruses can only infect dividing cells. NK cells also have a short half-life in vivo, yet expressing membrane bound IL-15 increases viability and cytotoxicity in NK cells transduced with retroviruses [126]. Vectors based on lentiviruses, a subclass of retroviruses, are more popular likely because they are relatively safer [61].

**Table 2:** Studies aimed at improving transduction efficiency in NK cells

Vector	Cells used for transduction	Transduction efficiency	Main findings	References
Lentivirus	Primary NK cells	29% (range 16–41%) with excellent viability	Multiplicity of infection (MOI) of 25 is optimal. Higher MOI results in higher transduction but lower viability. Transgene expression peaked at 5 days post-transduction and was detectable for 2 weeks	[222]
Lentivirus	Primary NK cells and the YTS NK cell line	98% in the YTS NK cell line, up to 80% in primary NK cells	Transduction efficiency was 20% when fresh primary NK cells were transduced without cytokines. The efficiency increased up to 20–50% when IL-2 plus IL-12 were added and 80% when PHA, a lectin, was added to the culture. The transduction efficiency remained stable in the presence of PHA for 10 days in culture	[223]
Lentivirus	Primary NK cells	Depending on cell type, BX795 addition increased transduction efficiency up to tenfold	Inhibition of TBK1/IKKε complex by BX795 significantly improved transduction efficiency. Lentivirus RNA is probably recognized by pathways involving TBK1/IKKε complex involved in anti-viral response pathways, which may also include RIG-I, MDA-5, and TLR3	[224]
Lentivirus	Primary NK cells (mice)	40%	IL-2 is not required for transduction	[225]
mRNA transfection and lentivirus	Primary NK cells, NK-92, and NK cells from UCB	In NK-92 (56% with mRNA, 26% with lentivirus), in primary NK (approximately 10% with mRNA), and in UCB (12% to 73% with lentivirus)	mRNA delivery was more effective than lentiviral transduction in transducing the NK-92 cell line (56% vs. 26%)	[226]
Lentivirus	NK-92, LNK, YT, and DERL7 cell lines	15% in NK-92 and 30–40% in LNK, YT, and DERL7 cell lines	Lentiviruses are more efficient than retroviruses that require multiple rounds of transduction	[121]
Retrovirus	Primary NK cells	27–47% on days 5–6 of the culture and 52–75% 21 days after initial culture	MOI of 10 yields highest transfection efficiency	[227]
Retrovirus	UCB NK cells	49%	NK cells expressing IL-15 were detectable up to a year after infusion	[68]
Retrovirus	UCB NK cells	66.6%	NK cells purified from UCB and transduced with iCaspase-9, IL-15, and a CD19-specific CAR showed significant toxicity toward CD19 + tumors	[83]

Alternative methods developed to avoid risks associated with the viral delivery of genes have not been fully successful. A transposon-based gene delivery system has been reported, but its design still needs improvement [61]. mRNA delivery of CARs into NK cells has relatively high success rates, but the transfection is only transient. CD19-CAR NK generated by trogocytosis avoids side effects of viral transduction. However, this approach also has remained at the experimental stage [127]. Using nucleofection or electroporation for gene delivery results in 1–90% transient transfection efficiency with viability ranging from 45 to 97%, suggesting that the technology is not stable or very reliable.

Several approaches and compounds including dextran, PHA, and BX795 have been tested to improve transduction efficiency in NK cells (Table 2). Dextran, a cationic polymer, significantly increases transduction efficiency in human and mice primary NK cells transduced with lentiviruses [128]. Magnetofection improves lentivirus-mediated gene delivery [129]. A modified 3D nanochannel-electroporation (NEP) method that can transfect 100–200 cells at a single-cell resolution has been developed [130]. The method involves precisely positioning cells on nanochannels allowing precise transfection. The method has shown nearly 70% transfection efficiency using CAR NK cells. However, this technology also delivers CARs only transiently.

#### Challenges in expanding CAR NK cells

A second significant challenge in CAR NK research is the limited expansion of NK cells in vitro and especially in vivo. Expanding CAR NK cells, especially PB NK cells, to clinical scale in vitro is difficult because a large number of NK cells are needed for immunotherapy and NK cells comprise only about 10% of the PB lymphocytes and therefore need to be expanded in culture [131]. Several methods have been developed to improve in vitro expansion rates in purified PB NK cells [90, 132–135]. A common method involves co-culturing PB NK cells with K562 feeder cells genetically modified to express IL-15 and the adhesion molecule 4-1BBL [90]. Telomere shortening seen in NK cells expanded by this technology can be prevented by expressing membrane bound IL-21 [135]. An approximate 2000-fold expansion of the NK cells from fresh or cryopreserved UCB in two weeks was achieved using these artificial antigen-presenting cells [136]. Expanded cells retain high expression levels of NKp44, NKG2D, TRAIL, and FasL, and their killing activity does not decrease compared to the fresh NK cells [137]. Nevertheless, NK cell exhaustion and loss of expansion capacity remain as concerns in long-term culturing exceeding 4 weeks.

Limited in vivo expansion of NK cells after infusion due to unknown reasons is an especially significant challenge. Studies using iPSCs show that in vivo expansion rates in CAR NK and CAR T cells are correlated with their anti-tumor activity [89]. IL-2 diphtheria toxin fusion protein (IL-2DT) infusion may help in vivo expansion of haploidentical NK cells in AML patients by inhibiting Tregs [138].

A recent phase 1/2 clinical trial has shown that incorporating IL-15 into anti-CD19 CAR NK cells results in significant improvement in in vivo expansion [68]. A UCB unit was thawed and cultured in the presence of K562 expressing IL-21 and 4-1BBL and IL-2. Cells were transduced with an anti-CD19 CAR NK vector expressing IL-15 and iCaspase-9 on day 6, expanded, and harvested for infusion on day 15. As noted above, eight out of the eleven (73%) patients had responses up to 13.8 months. Expansion, as measured by real-time PCR (RT-PCR) (copies of the viral genome per micrograms of genomic DNA), was detected three days after infusion and persisted for over a year. Expansion was seen only in patients that responded to the treatment.

#### Challenges in CAR NK cell therapy in solid tumors

The exciting results from Liu et al. observed in CAR NK cell therapy in liquid tumors [68] have not yet been seen in solid tumors possibly due to challenges such as solid tumor heterogeneity and the hostile tumor microenvironment (TME). CD19 in leukemia and lymphoma is highly and homogenously expressed on the surface of malignant B cells, and the CD19-CAR NK cells do not face substantial anatomical barriers in the blood compartment before establishing a contact with their targets. In solid tumors, however, the CAR NK cells must travel in bloodstream, migrate into the tissue, and establish a contact with the tumor cells while battling many inhibitory factors in the TME (Table 3).

**Table 3:** Challenges in building more efficient CAR NK cells for treatment of solid tumors and possible solutions^a^

Challenges	Possible solutions	References
Lack of available targets		
TAA expression not homogenous	Use of oncolytic viruses in combination therapy or bi-specific CARs	[116, 228]
Clonal evolution		
On-target, off-tumor effects		
Anatomical barriers		
Insufficient trafficking or infiltration	Intra-tumoral injection of CAR NK cells; focused ultrasound guided delivery of CAR NK cells into tumor; targeting tumor vasculature; CAR NK cells expressing a chemokine(s)	[149, 155]
	Locoregional injection, use of stronger co-stimulatory domains, lymph depletion, in vivo administration of chemokine or cytokine-expressing CARs	[146, 169]
Insufficient NK cell proliferation and/or activation in vivo		
Immunosuppressive TME	TGF-β inhibition through neutralizing antibodies or dominant negative receptors	[229]
	Use of cytokines such as IL-2, IL-15, IL-12, or IL-18 to simulate NK cell proliferation and activation	[68]
	Inhibition of checkpoint blockades using anti-PD1, anti-TIGIT, etc	[165]
Metabolic abnormalities	Metabolic stimulation through inhibition of CD73 or arginase	[162]
NKG2D inhibition by TME	NKG2D activation through histone deacetylase inhibitors	[160, 230]

^a^*TAA* tumor associated antigens, *TME* tumor microenvironment

CAR NK cell therapy seems more promising in breast, ovarian, and prostate cancers than other types of solid tumors because these tissues are easily and safely accessible and destruction of the normal tissue can be tolerated. In addition, specific antigen targets such as PSMA or prostate stem cell antigen (PSCA) are already available for targeting prostate cancer [139]. Indeed, transduction of YTS cell lines with DAP12-based anti-PSCA CAR induces strong phosphorylation of ZAP70 and fully eradicates tumors in a portion of the mice tested [140]. CAR NK-92 cells expressing EpCAM accumulate in prostate tumor and are highly cytotoxic against EpCAM+ prostate cancer cells even at low doses [141]. iPSCs-derived CAR-NK cells targeting mesothelin have shown to have similar efficacy similar to that of CAR-T cells but with fewer toxicities [89].

In breast cancer, NK-92 cells expressing a HER2-specific CAR enhance killing of human breast cancer cell lines and increase survival in mice breast and renal cell carcinoma (RCC) models [107]. CAR NK cells targeting EGFR derived from PB are effective in triple-negative breast cancer (TNBC) both in vitro and in vivo [142]. A recent study focused on tissue factor (TF) highly expressed in 50–85% of the TNBC patient tumors with a FVII as a ligand [143]. FVII-CAR NK-92MI cells expressing CD16 are effective in vivo and enhance tumor killing by TF-targeting antibody-like immunoconjugates (L-ICON), in which FVII was fiused to the Fc fraction of IgG1. Major challenges in the use of CAR NK cells for treatment of solid tumors can be categorized into the following groups:

##### Lack of available targets and antigen heterogeneity

The first step in designing CARs is to locate a target antigen highly and uniformly expressed on the tumor cell surface. In solid tumors, most TAAs are expressed by cells not only in tumor but also in vital organs, making it impossible to avoid “on-target, off-tumor” effects [144]. In addition, TAA expression may be dramatically different between single cell clones from the same tumor due to clonal evolution and downregulation of TAA expression by tumors to evade immune surveillance. Bispecific CARs, composed of two scFvs recognizing two different antigens on the same CAR and activated only when both antigens are engaged, have been successful in improving specificity in prostate cancer [145].

##### Anatomical barriers

Local injection, intra-peritoneal injection, and focused ultrasound-guided delivery of CARs into tissues have been experimented to overcome the anatomical barriers faced by the immune cells carrying CARs in solid tumors. In orthotopic models mimicking human pleural malignancies, pleural injection of CAR T cells is more effective and results in longer functional persistence than the *i.v.* injection [146]. Intrapleurally administered CAR T cells were able to circulate and persist in the periphery, suggesting that locally accessible sites may serve as “distribution centers” for CAR T therapy. Regional administration of CAR T cells was additionally useful in reducing the therapeutic dose of the CAR T cells. Endothelial cells in tumor vasculature have been targeted and successfully lysed by anti-PSMA-CAR T cells [147], as PSMA is expressed in neovasculature [148]. Anti-HER2 CAR NK-92 cells were delivered into the brain of mice with metastatic breast cancer using focused ultrasound [149]. The authors applied ultrasound bursts through intact skull combined with *i.v.* injection of microbubbles that temporarily allowed passage of NK-92 cells from the blood brain barrier into the brain without significant tissue damage.

##### Limited CAR NK cell trafficking and infiltration into tumor sites

The number of NK cells infiltrating into tumor has a prognostic value in solid tumors including gastric, lung, and colorectal cancers [150]. CARs that express chemokine receptors (CCRs) matching CC chemokine ligands (CCLs) expressed by the tumor cells acquire increased infiltration capabilities. Wennerberg et al. (2015) reported that in vitro expansion of NK cells was associated with a tenfold increase in CXCR3 expression compared to resting NK cells. Expanded cells migrated strongly toward CXCL10-transfected melanoma, but not CXCL10-negative tumor cells [151]. NK cells expressing CXCR2 show increased migration toward tumors expressing CXCR2 ligands [152]. The cells additionally show increased calcium mobilization, cytotoxicity, and adhesion properties. Overexpression of CXCR4 in anti-EGFRvIII CAR NK cells results in increased chemotaxis toward CXCL12/SDF-1α-secreting glioblastoma cells, complete tumor remission in a number of mice, and increased overall survival [153]. Oncolytic viruses are anti-tumorigenic and may also be anti-angiogenic through negative regulation of VEGF expression [154, 155]. In addition, oncolytic viruses induce an inflammatory immune response and enhance immune cell trafficking by modifying the TME [155]. Our group first explored the concept of combining anti-EGFR CAR NK cells and oncolytic virus therapy in a metastatic breast cancer animal model. The combination of EGFR-CAR NK-92 cells with oHSV-1 resulted in more efficient killing of breast cancer tumor cells engrafted into the brain compared to monotherapies and significantly improved survival of mice bearing breast cancer tumor cells in the brain [116].

##### Immunosuppression in the TME due to TGF-β

Tumors express immunosuppressive factors including TGF-β, IL-10, PD-1, or arginase. In vitro expanded NK cells are highly cytotoxic against cancer cells. However, they lose this ability upon injection in vivo. TGF-β is produced by neutrophils, macrophages, and Tregs in the TME. Tregs and immunosuppressive myeloid-derived suppressor cells (MDSCs) are actively recruited to the TME to create a strong immunosuppressive environment promoting tumor growth [156].

Several strategies to minimize inhibitory effects of TGF-β have been reported. Administration of TGF-β kinase inhibitors along with NK cells preserves the cytotoxic potential and expression of activating NK receptors NKG2D and CD16 [157]. Use of fresolimumab, a TGF-β neutralizing antibody, and galunisertib, an inhibitor of TGF-βRI, have been reported as safe and tolerable in solid tumors in humans [158]. Hybrid CARs with an extracellular TGF-β receptor domain linked to an intracellular NKG2D domain enhance anti-tumor activity in NK-92 cells and knocking down SMAD3, the downstream mediator of TGF-β, in NK cells improves cytotoxicity in solid tumors [75, 159]. UCB NK cells expressing a dominant-negative TGF-β receptor retain their capacity to secrete IFN-γ and kill glioblastoma cells in the presence of TGF-β. Entinostat, a narrow spectrum histone deacetylase inhibitor, increases MICA expression on tumor cells and NKG2D expression in primary NK cells even in hypoxic conditions, leading to enhancement of cytotoxicity of NK cells against tumor cells [160]. It enhances both tumor cell recognition and killing.

##### Immunosuppression in the TME due to metabolic disturbances

The TME additionally suffers from hypoxia and nutrient depletion leading to acidosis, which further suppresses immune responses [156]. Hypoxia supports tumor growth by inducing metabolic disturbances and increasing angiogenesis and expression of tumor growth factors. In addition, it downregulates expression of NK cell activating receptors NKp30, NKp46, NKp44, and NKG2D for optimal tumor growth and metastasis [161]. CD73 induces expression of arginase, an immunosuppressive metabolite. Arginase is produced under hypoxic conditions and inhibits NK cell functions. CD73 inhibition increases homing of NKG2D-CAR NK cells, which target tumor cells expressing NKG2D liagnds, to the tumor site and improves anti-tumor responses in animal models for lung cancer [162].

##### Immunosuppression in the TME due to checkpoints

Tumor cells evade immune surveillance through expression of checkpoint proteins that prevent immune cell activation. TIGIT inhibits NK cell cytotoxicity by opposing CD226 [163]. PD-1 + NK cells show reduced proliferation and effector functions, whereas PD-L1 + NK cells possess better effector functions [164]. Inhibition of PD-1 or PD-L1 using checkpoint blocking agents reactivates exhausted immune cells and achieves durable clinical responses [165]. Combining CARs with blockers of checkpoint proteins such as PD-1, CTLA-4, LAG3, and TIGIT has the potential to achieve persistent therapeutic benefits [145]. NK-92 cells engineered to express high-affinity CD16, IL-2, and a PD-L1-specific CAR contain high levels of perforin and granzyme and lyse human cancer cell lines including breast, lung, and gastric cancers [166].

### Efforts to build more efficient CARs

Although CAR NK cells and CAR T cells have opened a new era in immunotherapy, results from clinical studies suggest that improvement is still needed. Key areas that need further attention for improvement include modifying CAR structure for better efficiency and/or suitability to the TME, over-expressing activating and/or blocking inhibitory receptors on NK cells, improving cytotoxic mechanisms involving death receptors or granule-dependent pathways, and/or use of multiple compounds to increase CAR efficacy. Efforts to optimize CAR NK cell efficacy are extensive but can be categorized into the groups below:

#### CARs targeting multiple proteins

Some of the recent CAR NK-92 constructs include TCR-CARs created by fusing the extracellular domain of TCR (both TCRα and TCRβ) to a CAR-signaling tail composed of CD28 and CD3ζ signaling domains to enable the cells to recognize an array of targets, possibly some of the proteins presented on the MHC molecules, rather than a single TAA [167]. Expression of TCR-CAR in NK-92 cells grafts the cells with target recognition specificity of TCRs. This design has the potential of allowing the CAR NK-92 cells to target the proteome derived from tumor cells [167]. It would be interesting to determine whether this approach would work in primary NK cells or NK cells from other sources.

#### CAR-modified immune cells secreting enzymes, interleukins, or chemokines

“Armored” CAR design involves expressing cytokines, chemokines, or their receptors in CAR-modified immune cells to respond to threats from the TME or protect the CAR-bearing cells from immune suppression. CAR T cells expressing enzymes such as catalase, which can convert H_2_O_2_ into harmless by-products, or cytokines such as IL-15 have been developed [168]. CAR NK cells secreting autocrine IL-2 and IL-15 and silencing inhibitory KIRs have improved in vivo persistence [169]. CARs specific for various CC chemokines and/or their receptors have been reviewed above in the section on “Limited CAR trafficking and infiltration into tumor sites”.

#### Converter CARs

While some CARs provide the immune cells with more “armors” against the hostile tumor cells or the TME, others have been designed to “convert” the hostile actions into desirable effector functions. This “converter” design includes combining ecto- and endodomains of different molecules that graft specificity of the former coupled with the signaling capacity of the latter onto the immune cell. For example, CARs that combine ectodomain of IL-4 with the endodomain of IL-7 convert the inhibitory effects of IL-4 in the TME into the proliferative actions of IL-7 [170]. Similarly, CARs converting immunosuppressive effects of TGF-β into activating functions of NKG2D by fusing the extracellular and transmembrane portions of TGF-βRII and the intracellular domains of NKG2D, an activating receptor, have been reported [171]. Other variants of converter CARs include double chimeric design that involves introducing two CARs with different scFvs but the same intracellular signaling domain (dual-signaling CAR) [172]. A recent study sought to interfere with PD-1 signaling to reverse immunosuppression by PD-1 [173]. The authors fused the extracellular domain of PD-1, transmembrane and cytoplasmic domains of NKG2D, and the cytoplasmic domain of 4-1BB to create a chimeric co-stimulatory converting receptor-NK-92 cell line that reversed the inhibitory PD-1 signaling and increased anti-tumor activity against lung cancer cells [173].

#### Inducible CARs

A fourth-generation CAR, also called TRUCK, has been developed [172]. In this design, inducible IL-12 is expressed only after encounter with the tumor specific antigens. IL-12 release activates innate immune cells that kill tumor cells carrying the specific antigen targeted by CAR. A form of TRUCK activated by inducible HIF1α in the TME has been reported [61]. Inducible expression of CAR can also be achieved using an injectable drug. A version of inducible CARs also termed “trans-signaling CARs” has been developed. These designs are especially useful in increasing safety and specificity of CARs when multiple proteins or isoforms of the same protein are targeted. Such CARs may engage antigens when either of multiple isoforms is available to increase range of efficacy or may require binding to all isoforms of the target for activation for increased specificity [172].

#### Modification of CARs using the CRISPR/Cas9 system

Recent advances in CRISPR/Cas9 technology provide additional opportunities by enabling researchers to insert a CAR permanently into a desired position in the genome rather than insertion into random locations employed by most of the current viral transduction methods [174]. Another advantage of the CRISPR/Cas9 system is that it can be used to screen the whole genome in loss-of-function studies to reveal genomic regions or pathways important in mediating CAR toxicity. This approach has identified FADD and TNFRSF10B (TRAIL-R2) in CAR T cells but has not yet been studied in CAR NK cells [175]. Simultaneous administration of compounds to regulate these pathways can improve CAR effector functions.

The CRISPR/Cas9 technology holds great promise in advancing the CAR NK cell research through boosting effector functions, especially for iPSC-derived CAR NK cells [89]. It can be used to knock out several genes simultaneously [176]. It is especially advantageous to use in modifying NK cells due to the fact that NK cell activation is achieved through a balance between inhibitory and activating signals. This technology can knock-in activating while simultaneously knocking-out inhibitory signals. It is known that viral integration rates are higher in genomic regions with active transcription or highly accessible chromatin [177]. The CRISPR/Cas9 system can be used to insert a CAR cassette into these regions to generate stably transduced primary NK cells, improving both efficacy and safety of CAR NK cells [61]. CRISPR/Cas9 has already been used to “knock-in” a CAR while simultaneously “knocking-out” endogenous elements such as components of the TCR that may interfere with the CAR functions in CAR T cells [178, 179].

Only few CRISPR/Cas9 studies have been conducted using NK cells possibly because NK cells are more difficult to transduce [180]. Two studies that successfully transfected NK cells with Cas9 protein and target-site-specific gRNA or DNA with homology to sequences flanking DNA double strand breaks using electroporation have been reviewed elsewhere [180]. In one of those studies, 60% and 34% reduction at the mRNA and protein level, respectively, in TGF-βR II expression was achieved [181]. Another study improved post-transduction survival up to 80% while maintaining efficient reduction in *Ptprc* gene expression [182]. This study additionally revealed that both homo- and heterozygous deletion of the gene of interest are possible in the same reaction.

Despite the low number of studies reporting CAR NK cells created using the CRISPR/Cas9 technology, multiplex knockout of three genes (*TRAC*, *B2M*, and *PD-1*) in CD19-CAR T cells has already been achieved [183]. However, low knock-out rates for PD-1 were reported in that study. Another recent study was successful in efficiently knocking out PD-1 in CAR T cells targeting EGFRvIII [184]. The disruption did not change T cell phenotype or expression of other checkpoint receptors and enhanced activity of the CAR T cells. TGF-βR II has also been knocked out in CAR T cells, resulting in decreased T cell exhaustion [185].

#### Use of CARs along with monoclonal antibodies, nanoparticles, or chemical compounds for increased efficacy

NK cells can be used in tandem with antibodies, radiation, chemotherapy [186], romidepsin, a histone deacetylase inhibitor, nanoparticles [187, 188], and/or cabozantinib, a tyrosine kinase inhibitor [189]. Romidepsin improves efficacy of CD20-CAR NK cells [187], and cabozantinib enhances effects of CAR NK-92 directed against EGFR [189]. Use of CAR NK cells along with monoclonal antibodies widely used in cancer immunotherapy represents an interesting field of research. Bi- and tri-specific killer cell engagers (BiKEs and TriKEs) have been used to improve NK cell anti-tumor activity via the CD16 (FcγRIII) receptor on NK cells [190]. A majority of NK cells express CD16 that enables them to kill tumor cells opsonized by therapeutic monoclonal antibodies such as rituximab through ADCC [191]. An excellent review on potential of NK cells in combination therapy with monoclonal antibodies is available elsewhere [192].

#### CAR-engineered hematopoietic stem cells

CAR-engineered stem cells represent a viable option to eradicate viral infections or cancers as they can differentiate into CAR T cells or CAR NK cells. Hematopoietic stem/progenitor cell (HSPC) populations transfected with a CAR against HIV provide long-term immunity against HIV infections [193]. The CAR-stem cells differentiated into functional NK and T cells and suppressed HIV replication in vivo and in vitro*.* Since stem cells are self-renewing, these CAR-modified immune cells may lead to permanent remission [194]. This method has implications for transplantation studies and immunity against viral infections in addition to cancer immunotherapy. Protocols for generating NK cells through differentiation of genetically modified hematopoietic stem cells have already been developed [195].

#### Optimization of CAR signaling domain

Incorporation of 4-1BB, a surface protein expressed by NK and activated T-cells, improves NK cell cytotoxicity and IFNγ production [93, 94]. Although not expressed in NK cells, CD28 is commonly incorporated into CAR design for NK cells to amplify extracellular signaling. NK-92 cells carrying CARs with CD28/CD3ζ and 4-1BB/CD3ζ signaling domains have stronger cytotoxicity than CARs with only CD3ζ in killing HER2-expressing breast carcinoma cells [107]. Stimulation with 4-1BB is more effective than CD28 in expanding CD8+ T cells, and it has been shown that incorporation of both CD28 and 4-1BB co-stimulatory domains improves in vivo persistence in CAR T cells [196]. Additional potential co-stimulatory molecules being explored in CAR design for NK cells include 2B4 (CD224), DAP10, and DAP12 [94, 113, 140, 197].

Although 2B4 signaling alone only weakly induces NK cell activation, CARs including 2B4 along with CD3ζ enhance all aspects of the NK cell activation against leukemia and neuroblastoma cells [113]. Additionally, CARs utilizing a signaling domain for DAP12, which is involved in signal transduction of activating receptors such as NKG2C, NKp44, and KIR3DS1 [198, 199], exhibit improved NK cell cytotoxicity compared to CARs with CD3ζ as the sole signaling domain [140].

#### Manipulation of immunologic synapse formation and NK cell subsets

Immunologic synapse formation by CAR NK cells and possible differences in CAR production efficiency among different subtypes of NK cells have not been fully investigated in the literature. Synapse formation is a step-wise process that involves distribution, rearrangement and clustering of signaling molecules, F-actin, and tumor antigens at the site of contact. Synapse formation between NK cells and various target cells has been extensively investigated [200]. The NK IS formation involves central and peripheral supramolecular activation cluster (SMAC). The central and peripheral SMACs involve actions by distinct sets of proteins. The central domain includes mostly perforin, whereas the peripheral domain consists of surface receptors and F-actin. Upon stimulation, surface receptors including CD2, CD11a, CD11b, and F-actin rapidly accumulate at peripheral SMAC. This accumulation is dependent on actin polymerization driven by Wiskott–Aldrich syndrome protein. Accumulation of perforin at central SMAC is, on the other hand, slow and appears only after peripheral SMAC activation. The strong need for studying IS formation in CAR NK cells as well as critical steps of IS formation during the killing of target cells by NK cells has been reviewed elsewhere [201].

Similarities and differences between the conventional and CAR NK cell signaling events occurring at IS have not been well understood. A better understanding and possible manipulation of the IS formation can significantly improve CAR efficacy [201] because IS is critical for successful elimination of the target [202]. During natural T cell receptor activation, CD4/CD8 T cells interact with MHC molecules on antigen presenting cells (APCs) resulting in activation of Lck which then phosphorylates ZAP70, resulting in signal 1 for T cell activation. Signal 2 required for activation is a result of CD28 on T cells interacting with CD80/CD86 on APCs creating an IS with a 15-nm gap [203]. The 15-nm distance physically excludes other inhibitory molecules such as CD45 with large domains from entering into IS. It is unknown whether these processes occur similarly at IS formed between the CAR NK cells and their targets.

Studying different NK cell subsets carrying the same CAR may reveal subtypes more efficient than others for specific purposes [204]. It is estimated that there are 6000–30,000 different NK cell phenotypes in a healthy donor due to the high number of activating receptors with different functions expressed at different stages of development [138]. However, only limited data are available on the characteristics of these subtypes of NK cells.

#### Enhancement of CAR NK cell activation and proliferation by cytokines

NK cells in patients with tumors are often functionally impaired and live shorter than those in healthy individuals. Cytokines, or their analogs with longer in vivo half-life, can be used to increase NK cell activation, proliferation, trafficking, and persistence. Effects of several cytokines including IL-2, IL-12, IL-15, IL-18, and IL-21 on NK cell function have been reviewed in detail elsewhere [75]. The cytokines can be administered in vivo or used for in vitro stimulation before adoptive cell transfer. NK cells require IL-2 or IL-15 for optimal growth. NK-92MI, an NK-92 cell line constitutively expressing IL-2, has been generated [114, 143]. IL-2 and IL-15 are both included in the γ chain family of cytokines and share some common functions [114]. Co-expressing IL-15 and an EpCAM CAR in NK-92 cells resulted in a predominant intracellular acculmulation of the cytokine and selection of CAR-bearing cells upon IL-2 withdrawal, which eliminated all but the transduced NK-92 cells. These CAR NK cells successfully lysed breast cancer cells in the absence of any other cytokine [114].

IL-2 is safe to administer in lung cancer patients and induces proliferation and cytotoxic capacity in NK cells, but it also attracts and stimulates immunosuppressive Tregs at high doses [205]. IL-15 may be more effective in improving NK cell function because it is anti-neoplastic, more potent than IL-2, and does not attract Tregs. CAR NK cells may be stimulated by IL-15 through co-culturing with feeder K562 cells expressing membrane-bound IL-15 and 4-1BB ligand [94]. Co-culturing PB NK cells with K562-based feeder cells expressing membrane-bound IL-21 significantly improves NK cell expansion in vitro. IL-12 at the dose of 500 ng/kg/day was initially found safe in a phase 1 clinical trial. However, the same dose resulted in serious side effects in 12 of 17 patients enrolled in the study and two patients died, leading to a ban by the FDA on all clinical trials involving IL-12 [206].

#### CAR NK activation and proliferation through manipulation of NK cell receptors

NK cell effector functions can be improved through inhibiting inhibitory KIRs such as KIR2DL-1, -2, or -3 and/or overexpressing activating KIRs. Inhibitory receptors (as listed by Pockley et al*.* [205] with their ligands on tumor indicated in parentheses) include PD-1 (PD-L1), KIRs (HLA-C), LAG3 (MHC II), NKG2A/CD94 complex (HLA-E), TIGIT (CD155), TIM3 (Galectin-9), and KLRG-1 (E-cadherin), whereas activating NK receptors include NKG2D (MICA/B), KIRs (HLA-C), CD226 or DNAM-1 (CD112 or Nectin-2, CD155 or PVR), and NKG2C/CD94 complex (HLA-E and Hsp70). In addition, receptors 2B4 (CD244), CS1 (CD319), and LLT1 (CLEC2D) have been identified on NK cells. 2B4 and CS1 regulate NK cell cytotoxicity, whereas LLT1 is additionally expressed in prostate and TNBC cells and plays a role in evading NK cell-mediated tumor cell killing [207].

One of the most studied NK cell receptors is NKG2D. Histone deacetylase inhibitors such as sodium valproate enhance NK-mediated cell killing by increasing expression of NKG2D ligands MHC class I chain-related molecules MICA and MICB on tumor cells [208]. Parihar et al*.* (2019) created CAR NK cells expressing a chimeric receptor in which NKG2D is fused to CD3ζ [209]. These CAR NK cells reduced tumor burden through killing intra-tumoral MDSCs that expressed high levels of NKG2D ligands and, unlike unmodified NK cells, released cytokines even in the TME. The CAR NK cells additionally improved CAR T cell trafficking to the tumor site through chemokine secretion. The authors concluded that these CAR NK cells should be used in combination with CAR T cells for optimum efficacy in the TME in solid tumors.

Use of anti-KIR antibodies such as lirilumab to enhance in vivo CAR NK effector functions represents a novel approach in the treatment of solid tumors. Lirilumab is a fully human IgG4 mAb [210] that improves NK cell anti-tumor functions by inhibiting KIR2D subgroup [211] receptors on NK cells and blocking the repressive KIR-MHC interactions [210]. Lirilumab efficacy has been tested in enhancing NK effector functions in both liquid and solid tumors [210]. Lirilumab, in combination with ipilimumab, is being tested for treatment of advanced solid tumors in a clinical trial (NCT01750580), but the trial is still recruiting patients [212]. In vitro evidence suggests that lirilumab enhances killing of head and neck squamous cell carcinoma (HNSCC) cells by NK cells [213]. Enhancement of anti-tumoral effects of lirilumab, in combination with rituximab, has been well demonstrated in hematologic malignancies both in vivo and in vitro [214]. In myelodysplastic syndrome patients, lirilumab is effective both as a monotherapy and in combination with azacitidine although the authors cautioned that further studies are needed to confirm their findings [215]. IPH2101, a prior version of lirilumab that recognizes the same epitope, has also been tested in the clinic [216–218]. It will be interesting to determine if abrogation of KIR inhibitory signaling by KIR antibodies will further improve anti-tumor activity of CAR NK cells.

#### Modifying cell metabolism to improve CAR NK effector functions

Enhancing CAR NK cell activity in solid tumors through modulation of tumor metabolism has received little attention in the literature. Extracellular adenosine generated from ATP present in the hypoxic tumor environment through actions of CD39 and CD73 plays critical roles in immune evasion, inhibition of NK cell maturation, and suppression of NK cell trafficking to the tumor site [162]. Adenosine inhibition using anti-CD39 and anti-CD73 antibodies improves targeted therapy in ovarian cancer [219]. CD73 is highly expressed in several solid tumors including glioblastoma, prostate, and lung cancer, and its inhibition using neutralizing antibodies further improves therapeutic effects of NKG2D-engineered CAR NK cells in a mice model of lung cancer [162].

### CAR NK cell clinical trials

Currently, there are 19 CAR NK clinical trials available on *ClinicalTrials.Gov* website (Table 4). Eleven and eight trials have targeted liquid and solid tumors, respectively. The most common targets (with number of clinical trials in parentheses) are CD19 (7), ROBO1 (3), and CD22 (2). One clinical trial has been completed, eight have been recruiting patients, one is suspended, and one is withdrawn. Five trials have not begun recruiting patients, and the status of the remaining three trials is unknown. Five trials are at early phase 1, four at phase 1, and ten are at phase 1 or 2 stages. Fourteen of the trials originated from China, three from the USA, one from Singapore, and one from Germany.

**Table 4:** Current CAR NK clinical trials listed on *ClinicalTrials.Gov*

Tumor type	Tumor type	Target antigen	NCT number	Types of NK cells	Status	Phase	Country
ALL	Liquid	CD19	NCT00995137	Haploidentical donor NK cells	Completed	1	USA
Solid tumors	Solid	NKG2D ligands	NCT03415100	Autologous or allogeneic CAR NK cells targeting NKG2D ligands	Recruiting	1	China
B-lymphoid malignancy, ALL, CLL, lymphoma	Liquid	CD19	NCT03056339	UCB NK cells	Recruiting	1/2	USA
Multiple myeloma	Liquid	BCMA	NCT03940833	NK-92 cells	Recruiting	1/2	China
Solid tumors	Solid	ROBO1	NCT03940820	Unclear	Recruiting	1/2	China
Pancreatic cancer	Solid	ROBO1	NCT03941457	Unclear	Recruiting	1/2	China
Solid tumors	Solid	ROBO1	NCT03931720	Unclear	Recruiting	1/2	China
Glioblastoma	Solid	HER2	NCT03383978	NK-92 cells	Recruiting	1	Germany
B-cell acute lymphoblastic leukemia	Liquid	CD19	NCT01974479	Haploidentical donor NK cells	Suspended	1	Singapore
AML	Liquid	CD33	NCT02944162	NK-92 cells	Recruiting	1/2	China
Mantle cell, follicular, and B-cell lymphoma	Liquid	CD19	NCT03579927	UCB NK cells	Withdrawn	1/2	USA
Refractory B-cell lymphoma	Liquid	CD22	NCT03692767	Unclear	Not yet recruiting	Early 1	China
Refractory B-cell lymphoma	Liquid	CD19	NCT03690310	Unclear	Not yet recruiting	Early 1	China
Ovarian cancer	Solid	Mesothelin	NCT03692637	Unclear	Not yet recruiting	Early 1	China
Prostate cancer	Solid	PSMA	NCT03692663	Unclear	Not yet recruiting	Early 1	China
Refractory B-cell lymphoma	Liquid	CD19, CD22	NCT03824964	Unclear	Not yet recruiting	Early 1	China
ALL, CLL, mantle cell and follicular lymphoma	Liquid	CD19	NCT02892695	NK-92 cells	Unknown	1/2	China
ALL, T-cell leukemia and lymphoma	Liquid	CD7	NCT02742727	NK-92 cells	Unknown	1/2	China
Hepatic, lung, pancreas, breast, colon, and gastric cancer	Solid	MUC1	NCT02839954	Unclear	Unknown	1/2	China

In one of those studies, a phase 1/2 trial (NCT02944162) targeting CD33 in AML patients had three patients who were administered nearly five billion irradiated CD33-CAR NK-92 cells. Although therapeutic response was limited, the patients did not show any significant adverse reactions after administration of up to five billion NK-92 cells, suggesting that injection of a large number of NK-92 cells may be safe [67]. A recent promising phase 1/2 clinical trial in the USA (NCT03056339) is being conducted to investigate the safety and efficacy of primary NK cells obtained from UCB carrying a CD19-CD28-CD3ζ-2A-iCasp9-IL15 construct in patients with relapsed/refractory B-cell ALL, CLL, and non-Hodgkin lymphoma. The CAR NK treatment was carried out in conjunction with lymphodepleting chemotherapy. Very encouraging results from this trial have recently been published [68]. As summarized above, eight of the 11 patients responded to the therapy. Importantly, NK cells persisted in patients for over 12 months possibly due to the incorporation of IL-15 in the CAR vector. A clinical trial being conducted in Germany has been investigating the safety and tolerability of NK-92 cells carrying anti-HER2 CAR in glioblastoma patients (NCT03383978).

## Conclusions

CAR-engineered T cells have already achieved impressive responses in treating some hematologic malignancies. NK cells offer an additional advantage in that they may not require any immunological matching between donor and patients and thus have the potential for developing “off-the-shelf” products for cancer immunotherapy. Although some challenges still exist, the recent success involving CD19-CAR NK cells expressing IL-15 [68] suggests that a broader application and advancement in using “off-the-shelf” CAR NK cell products for the treatment of hematologic malignancies and solid tumors may occur in the near future.

## Data Availability

Not applicable.

## Abbreviations

ADCC: Antibody-dependent cellular cytotoxicity
ALL: Acute lymphoblastic leukemia
AML: Acute myeloid leukemia
APC: Antigen presenting cell
CAR: Chimeric antigen receptor
CCL: CC chemokine ligand
CCR: CC chemokine receptor
CLL: Chronic lymphocytic leukemia
CR: Complete remission
CRi: Complete remission with incomplete hematologic recovery
CRS: Cytokine release syndrome
CSC: Cancer stem cell-like cell
DLBCL: Diffuse large B-cell lymphoma
FasL: Fas ligand
FDA: US Food and Drug Administration
GM-CSF: Granulocyte-macrophage colony stimulating factor
GvHD: Graft vs. host disease
HLA: Human leukocyte antigen
HNSCC: Head and neck squamous cell carcinoma
HSPC: Hematopoietic stem/progenitor cell
IFN-γ: Interferon-γ
IL-2DT: IL-2 diphtheria toxin fusion protein
ILC: Innate lymphoid cell
iPSC: Induced pluripotent stem cell
IS: Immunologic synapse
ITAM: Immunoreceptor tyrosine-based activation motif
ITT: Intent-to-treatment
KIR: Killer immunoglobulin receptor
L-ICON: Antibody-like immunoconjugates
MDSC: Myeloid-derived suppressor cell
MM: Multiple myeloma
MOI: Multiplicity of infection
NCR: Natural cytotoxicity receptor
NEP: 3D nanochannel-electroporation
NK: Natural killer
NSG: NOD scid gamma
oHSV-1: Herpes simplex virus 1-based oncolytic virus
ORR: Overall remission rate
PB: Peripheral blood
PSMA: Prostate-specific membrane antigen
PSCA: Prostate stem cell antigen
RAG: Recombination activating gene
RCC: Renal cell carcinoma
RT-PCR: Real-time PCR
scFv: Single-chain antibody variable fragment
SMAC: Supramolecular activation cluster
TAA: Tumor-associated antigen
TCR: T cell receptor
TF: Tissue factor
TGF-β: Transforming growth factor-beta
TME: Tumor microenvironment
TNBC: Triple-negative breast cancer
TRAIL: TNF-related apoptosis-inducing ligand
UCB: Umbilical cord blood
